# Benzyl 3-dehydr­oxy-1,2,5-oxadiazolo[3′,4′:2,3]oleanolate

**DOI:** 10.1107/S1600536809026695

**Published:** 2009-07-15

**Authors:** Jun Hu, Xiaoyun Gong, Ruji Wang, Yong Ju

**Affiliations:** a Key Laboratory of Bioorganic Phosphorus Chemistry & Chemical Biology, Ministry of Education, Department of Chemistry, Tsinghua University, Beijing 100084, People’s Republic of China

## Abstract

The title compound, C_37_H_50_N_2_O_3_, is a benzyl ester derivative of oleanolic acid, a penta­cyclic triterpene, with a five-membered oxadiazole ring fused to the ring *A*. The triterpene *A* and *C* rings adopt slightly distorted half-chair conformations, whereas the remaining three six-membered rings are in chair forms.

## Related literature

For information on oleanolic acid and its derivatives, see: Chen *et al.* (2006 ▶); Liu (2005 ▶).
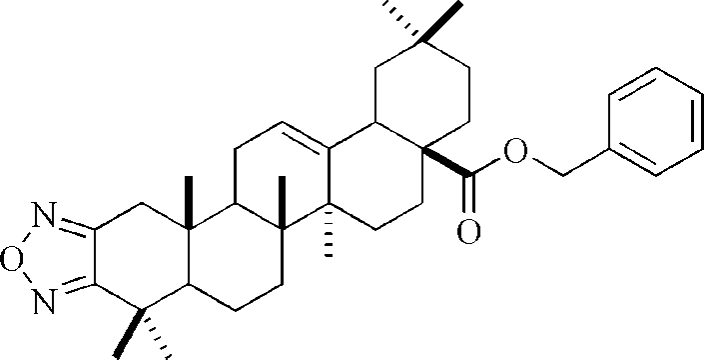


## Experimental

### 

#### Crystal data

C_37_H_50_N_2_O_3_
*M* *_r_* = 570.79
Orthorhombic, 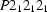

*a* = 8.7124 (19) Å
*b* = 19.054 (3) Å
*c* = 19.480 (3) Å
*V* = 3233.7 (10) Å^3^
*Z* = 4
Mo *K*α radiation
μ = 0.07 mm^−1^
*T* = 295 K
0.4 × 0.3 × 0.2 mm

#### Data collection

Bruker P4 diffractometer
Absorption correction: none
4315 measured reflections
3398 independent reflections
1649 reflections with *I* > 2σ(*I*)
*R*_int_ = 0.060
3 standard reflections every 97 reflections intensity decay: none

#### Refinement

*R*[*F* ^2^ > 2σ(*F* ^2^)] = 0.054
*wR*(*F* ^2^) = 0.131
*S* = 1.00
3398 reflections
381 parameters
1 restraint
H-atom parameters constrained
Δρ_max_ = 0.16 e Å^−3^
Δρ_min_ = −0.22 e Å^−3^

### 

Data collection: *XSCANS* (Bruker, 1997 ▶); cell refinement: *XSCANS*; data reduction: *XSCANS*; program(s) used to solve structure: *SHELXTL* (Sheldrick, 2008 ▶); program(s) used to refine structure: *SHELXTL*; molecular graphics: *SHELXTL*; software used to prepare material for publication: *SHELXTL*.

## Supplementary Material

Crystal structure: contains datablocks I, global. DOI: 10.1107/S1600536809026695/gk2215sup1.cif

Structure factors: contains datablocks I. DOI: 10.1107/S1600536809026695/gk2215Isup2.hkl

Additional supplementary materials:  crystallographic information; 3D view; checkCIF report

## Figures and Tables

**Fig. 1.**
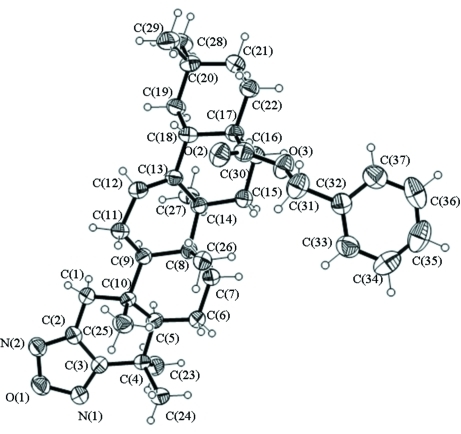
Molecular structure of the title compound with 35% probability displacement ellipsoids, showing the atomic numbering scheme.

## supplementary crystallographic information

### Comment

Recently, our group has designed and synthesized a novel series of oleanolic acid derivatives by chemical modification. Oleanolic acid is a pentacyclic triterpenoid with interesting biological activities that has been isolated from many medicinal plants (Liu, 2005). As our studies show the title compound, first reported by Chen *et al.* (2006), inhibits considerably growth of HepG-2 tumour cells and therefore its crystal structure is reported here.

The title compound was obtained as colourless plates. A view of the molecular structure with the numbering scheme is shown in Fig. 1. The molecule is composed of five fused six-membered rings, a five-membered heterocyclic ring and an independent benzene ring, *viz*. A(C1–C5/C10), B(C5–C10), C(C8/C9/C11–C14), D(C13–C18), E(C17–C22), F(C2—N2—O2—N1—C3) and G(C32–C37). The rings B, D and E adopt chair conformations, while the rings A and C adopt a slightly distorted half-chair conformation as a result of the presence of endo or exo double bonds. The lengths of double bonds N1–C3 and N2–C2 are nearly equal, even though they are in non-symmetrical environment.

### Experimental

Benzyl 2,3-dihydroxyiminooleanolate (500 mg, 0.85 mmol), 18-crown-6 (30 mg, 0.11 mmol) and t-BuOK (190 mg, 1.70 mmol) were dissolved in 15 ml tetrahydrofurane and 15 ml dichloromethane. The mixture was stirred for 10 min at 50°C. The product was treated with hydrochloric acid, then washed with water and extracted with ethyl acetate. The combined organic phase was washed with brine, dried and evaporated. Purification by flash chromatography (dichloromethane:methanol =100:1) gave title compound (456 mg; yield 94%). Crystals suitable for X-ray structure analysis were obtained *via* slow evaporation of a chloroform/acetone solution ( 1:5 vol.) at room temperature.

### Refinement

In the absence of significant anomalous scattering Friedel pairs were merged. The enantiomer has been assigned by reference to  unchanging chiral centers in the synthetic procedure. All hydrogen atoms were generated geometrically with C—H bonds of 0.93–0.98 Å according to criteria described in the *SHELXTL* manual (Bruker, 1997a). They were included in the refinement with U_iso_(H) = 1.2U_eq_(C), except methyl groups where U_iso_(H) = 1.5U_eq_(C).

### Crystal data

**Table:**

C_37_H_50_N_2_O_3_	*F*(000) = 1240
*M**_r_* = 570.79	*D*_x_ = 1.172 Mg m^−^^3^
Orthorhombic, *P*2_1_2_1_2_1_	Mo *K*α radiation, λ = 0.71073 Å
Hall symbol: P 2ac 2ab	Cell parameters from 49 reflections
*a* = 8.7124 (19) Å	θ = 2.1–15.1°
*b* = 19.054 (3) Å	µ = 0.07 mm^−^^1^
*c* = 19.480 (3) Å	*T* = 295 K
*V* = 3233.7 (10) Å^3^	Prism, colorless
*Z* = 4	0.4 × 0.3 × 0.2 mm

### Data collection

**Table:**

Bruker P4 diffractometer	*R*_int_ = 0.060
Radiation source: fine-focus sealed tube	θ_max_ = 25.5°, θ_min_ = 2.1°
graphite	*h* = −1→10
ω scans	*k* = −23→1
4315 measured reflections	*l* = −1→23
3398 independent reflections	3 standard reflections every 97 reflections
1649 reflections with *I* > 2σ(*I*)	intensity decay: none

### Refinement

**Table:**

Refinement on *F*^2^	Secondary atom site location: difference Fourier map
Least-squares matrix: full	Hydrogen site location: inferred from neighbouring sites
*R*[*F*^2^ > 2σ(*F*^2^)] = 0.054	H-atom parameters constrained
*wR*(*F*^2^) = 0.131	*w* = 1/[σ^2^(*F*_o_^2^) + (0.001*P*)^2^ + 1.8*P*] where *P* = (*F*_o_^2^ + 2*F*_c_^2^)/3
*S* = 1.00	(Δ/σ)_max_ = 0.008
3398 reflections	Δρ_max_ = 0.16 e Å^−^^3^
381 parameters	Δρ_min_ = −0.22 e Å^−^^3^
1 restraint	Extinction correction: *SHELXTL* (Sheldrick, 2008), Fc^*^=kFc[1+0.001xFc^2^λ^3^/sin(2θ)]^-1/4^
Primary atom site location: structure-invariant direct methods	Extinction coefficient: 0.00114 (8)

### Special details

**Table:**

Experimental. absolute configuration has not been established by anomalous dispersion effects in diffraction measurements on the crystal. The enantiomer has been assigned by reference to unchanging chiral centres in the synthetic procedure. The Friedel pairs were averaged in the final refinement because the Mo Kα radiation was used and the compound was made of C, H, N and O.
Geometry. All esds (except the esd in the dihedral angle between two l.s. planes) are estimated using the full covariance matrix. The cell esds are taken into account individually in the estimation of esds in distances, angles and torsion angles; correlations between esds in cell parameters are only used when they are defined by crystal symmetry. An approximate (isotropic) treatment of cell esds is used for estimating esds involving l.s. planes.
Refinement. Refinement of *F*^2^ against ALL reflections. The weighted *R*-factor *wR* and goodness of fit *S* are based on *F*^2^, conventional *R*-factors *R* are based on *F*, with *F* set to zero for negative *F*^2^. The threshold expression of *F*^2^ > σ(*F*^2^) is used only for calculating *R*-factors(gt) *etc*. and is not relevant to the choice of reflections for refinement. *R*-factors based on *F*^2^ are statistically about twice as large as those based on *F*, and *R*- factors based on ALL data will be even larger.

### Fractional atomic coordinates and isotropic or equivalent isotropic displacement parameters (Å^2^)

**Table:**

	*x*	*y*	*z*	*U*_iso_*/*U*_eq_	
O1	1.1911 (2)	0.29241 (10)	0.64263 (10)	0.0744 (7)	
O2	0.4830 (2)	0.57882 (10)	0.26204 (9)	0.0712 (6)	
O3	0.2427 (2)	0.55498 (10)	0.29343 (10)	0.0716 (6)	
N1	1.0434 (3)	0.26443 (11)	0.64843 (12)	0.0666 (8)	
N2	1.1866 (3)	0.35735 (12)	0.60900 (12)	0.0664 (8)	
C1	0.9740 (3)	0.42957 (13)	0.55969 (15)	0.0616 (9)	
H1A	1.0469	0.4475	0.5264	0.074*	
H1B	0.9514	0.4667	0.5922	0.074*	
C2	1.0416 (3)	0.36797 (13)	0.59652 (13)	0.0537 (8)	
C3	0.9526 (3)	0.31095 (13)	0.62036 (14)	0.0538 (8)	
C4	0.7825 (3)	0.30445 (13)	0.61483 (14)	0.0551 (9)	
C5	0.7189 (3)	0.37107 (12)	0.57597 (13)	0.0513 (8)	
H5A	0.7044	0.4064	0.6119	0.062*	
C6	0.5586 (3)	0.35847 (13)	0.54702 (15)	0.0601 (9)	
H6A	0.4963	0.3344	0.5809	0.072*	
H6B	0.5654	0.3289	0.5066	0.072*	
C7	0.4830 (3)	0.42843 (13)	0.52804 (14)	0.0584 (9)	
H7A	0.4705	0.4562	0.5694	0.070*	
H7B	0.3815	0.4191	0.5097	0.070*	
C8	0.5739 (3)	0.47144 (12)	0.47542 (13)	0.0486 (8)	
C9	0.7459 (3)	0.47566 (12)	0.49632 (13)	0.0478 (8)	
H9A	0.7481	0.5077	0.5357	0.057*	
C10	0.8244 (3)	0.40676 (12)	0.52265 (14)	0.0488 (8)	
C11	0.8355 (3)	0.51355 (12)	0.44024 (14)	0.0544 (8)	
H11A	0.9320	0.5299	0.4593	0.065*	
H11B	0.8593	0.4804	0.4040	0.065*	
C12	0.7521 (3)	0.57467 (13)	0.40973 (13)	0.0535 (8)	
H12A	0.8076	0.6033	0.3801	0.064*	
C13	0.6062 (3)	0.59207 (12)	0.42089 (13)	0.0476 (8)	
C14	0.5073 (3)	0.54920 (12)	0.47100 (13)	0.0482 (8)	
C15	0.3371 (3)	0.54676 (13)	0.44892 (15)	0.0582 (9)	
H15A	0.3245	0.5099	0.4150	0.070*	
H15B	0.2752	0.5344	0.4885	0.070*	
C16	0.2764 (3)	0.61563 (12)	0.41882 (13)	0.0555 (9)	
H16A	0.1717	0.6086	0.4034	0.067*	
H16B	0.2749	0.6510	0.4546	0.067*	
C17	0.3727 (3)	0.64233 (13)	0.35880 (14)	0.0527 (8)	
C18	0.5388 (3)	0.65564 (12)	0.38321 (13)	0.0528 (8)	
H18A	0.6014	0.6630	0.3420	0.063*	
C19	0.5519 (3)	0.72292 (12)	0.42714 (13)	0.0573 (9)	
H19A	0.5003	0.7151	0.4706	0.069*	
H19B	0.6594	0.7316	0.4369	0.069*	
C20	0.4830 (4)	0.78871 (12)	0.39294 (14)	0.0590 (9)	
C21	0.3161 (3)	0.77305 (13)	0.37401 (14)	0.0595 (9)	
H21A	0.2578	0.7648	0.4156	0.071*	
H21B	0.2724	0.8137	0.3512	0.071*	
C22	0.3016 (3)	0.70920 (13)	0.32701 (14)	0.0590 (9)	
H22A	0.3523	0.7192	0.2837	0.071*	
H22B	0.1939	0.7007	0.3175	0.071*	
C23	0.7150 (4)	0.30364 (15)	0.68788 (14)	0.0743 (10)	
H23A	0.7526	0.2633	0.7122	0.112*	
H23B	0.6050	0.3015	0.6853	0.112*	
H23C	0.7451	0.3455	0.7117	0.112*	
C24	0.7422 (4)	0.23391 (13)	0.57912 (16)	0.0779 (11)	
H24A	0.7836	0.2337	0.5334	0.117*	
H24B	0.6327	0.2287	0.5770	0.117*	
H24C	0.7853	0.1957	0.6048	0.117*	
C25	0.8705 (3)	0.35522 (13)	0.46425 (14)	0.0644 (9)	
H25A	0.7800	0.3394	0.4408	0.097*	
H25B	0.9233	0.3156	0.4835	0.097*	
H25C	0.9368	0.3788	0.4323	0.097*	
C26	0.5545 (3)	0.43558 (13)	0.40356 (13)	0.0616 (9)	
H26A	0.4478	0.4259	0.3956	0.092*	
H26B	0.6115	0.3925	0.4027	0.092*	
H26C	0.5922	0.4664	0.3684	0.092*	
C27	0.5144 (3)	0.58773 (12)	0.54287 (12)	0.0602 (9)	
H27A	0.4732	0.6342	0.5384	0.090*	
H27B	0.6191	0.5904	0.5580	0.090*	
H27C	0.4551	0.5619	0.5758	0.090*	
C28	0.4855 (4)	0.85000 (13)	0.44522 (15)	0.0815 (11)	
H28A	0.5897	0.8606	0.4573	0.122*	
H28B	0.4296	0.8367	0.4857	0.122*	
H28C	0.4386	0.8907	0.4251	0.122*	
C29	0.5774 (4)	0.81142 (14)	0.33019 (15)	0.0767 (11)	
H29A	0.5788	0.7741	0.2971	0.115*	
H29B	0.6805	0.8219	0.3442	0.115*	
H29C	0.5319	0.8525	0.3101	0.115*	
C30	0.3771 (3)	0.58947 (14)	0.30003 (14)	0.0593 (9)	
C31	0.2337 (4)	0.50425 (15)	0.23783 (15)	0.0753 (10)	
H31A	0.3175	0.4709	0.2410	0.090*	
H31B	0.2399	0.5278	0.1938	0.090*	
C32	0.0835 (3)	0.46753 (14)	0.24481 (14)	0.0614 (9)	
C33	0.0760 (4)	0.39893 (15)	0.26336 (16)	0.0816 (12)	
H33A	0.1668	0.3744	0.2708	0.098*	
C34	−0.0612 (4)	0.36458 (17)	0.27150 (17)	0.0924 (13)	
H34A	−0.0634	0.3174	0.2837	0.111*	
C35	−0.1919 (4)	0.40016 (18)	0.26151 (17)	0.0987 (13)	
H35A	−0.2849	0.3771	0.2682	0.118*	
C36	−0.1941 (4)	0.4695 (2)	0.24168 (16)	0.0971 (13)	
H36A	−0.2858	0.4930	0.2334	0.116*	
C37	−0.0519 (4)	0.50313 (16)	0.23452 (15)	0.0804 (11)	
H37A	−0.0490	0.5504	0.2226	0.096*	

### Atomic displacement parameters (Å^2^)

**Table:**

	*U*^11^	*U*^22^	*U*^33^	*U*^12^	*U*^13^	*U*^23^
O1	0.0625 (13)	0.0800 (13)	0.0808 (13)	0.0098 (12)	−0.0046 (12)	0.0181 (12)
O2	0.0653 (12)	0.0774 (12)	0.0710 (12)	−0.0048 (12)	0.0123 (12)	−0.0125 (11)
O3	0.0688 (13)	0.0741 (11)	0.0718 (12)	−0.0144 (13)	0.0066 (12)	−0.0206 (11)
N1	0.0565 (15)	0.0613 (13)	0.0820 (16)	0.0021 (14)	−0.0040 (15)	0.0038 (14)
N2	0.0561 (14)	0.0712 (14)	0.0720 (15)	0.0048 (14)	0.0006 (15)	0.0129 (14)
C1	0.0469 (15)	0.0509 (14)	0.087 (2)	−0.0029 (15)	−0.0043 (17)	0.0110 (16)
C2	0.0470 (16)	0.0578 (15)	0.0564 (16)	0.0052 (15)	0.0004 (15)	−0.0019 (14)
C3	0.0513 (16)	0.0497 (14)	0.0603 (17)	−0.0033 (15)	−0.0011 (16)	0.0004 (14)
C4	0.0536 (16)	0.0426 (13)	0.0691 (18)	−0.0012 (14)	−0.0076 (17)	0.0090 (15)
C5	0.0541 (16)	0.0429 (13)	0.0570 (16)	−0.0053 (14)	−0.0005 (15)	0.0004 (13)
C6	0.0482 (16)	0.0533 (15)	0.0789 (19)	−0.0037 (15)	−0.0041 (17)	0.0129 (16)
C7	0.0508 (16)	0.0536 (15)	0.0708 (18)	−0.0008 (15)	−0.0001 (17)	0.0126 (15)
C8	0.0447 (15)	0.0458 (13)	0.0553 (15)	−0.0034 (14)	0.0071 (14)	−0.0011 (13)
C9	0.0456 (14)	0.0447 (13)	0.0531 (15)	0.0027 (14)	0.0036 (14)	−0.0006 (13)
C10	0.0445 (15)	0.0405 (13)	0.0613 (16)	−0.0005 (13)	0.0034 (15)	0.0068 (13)
C11	0.0519 (16)	0.0456 (14)	0.0656 (17)	0.0032 (14)	0.0037 (16)	0.0070 (14)
C12	0.0586 (17)	0.0491 (14)	0.0530 (15)	−0.0001 (16)	0.0016 (16)	0.0063 (14)
C13	0.0518 (16)	0.0414 (13)	0.0498 (15)	0.0030 (14)	0.0026 (15)	0.0009 (13)
C14	0.0468 (15)	0.0438 (13)	0.0540 (15)	0.0008 (13)	0.0081 (15)	0.0018 (13)
C15	0.0487 (16)	0.0581 (16)	0.0679 (17)	0.0012 (15)	0.0051 (16)	0.0062 (16)
C16	0.0557 (17)	0.0541 (15)	0.0566 (16)	0.0067 (16)	−0.0029 (16)	0.0062 (14)
C17	0.0530 (16)	0.0514 (14)	0.0537 (16)	0.0034 (15)	0.0049 (15)	0.0054 (14)
C18	0.0619 (18)	0.0429 (13)	0.0536 (16)	0.0046 (15)	−0.0007 (16)	0.0061 (13)
C19	0.0622 (18)	0.0486 (14)	0.0609 (17)	0.0049 (16)	−0.0081 (17)	0.0037 (14)
C20	0.072 (2)	0.0411 (13)	0.0638 (17)	−0.0008 (16)	0.0027 (18)	−0.0011 (14)
C21	0.0629 (18)	0.0579 (16)	0.0578 (17)	0.0085 (17)	−0.0036 (17)	0.0028 (15)
C22	0.0648 (19)	0.0524 (15)	0.0598 (16)	0.0023 (17)	−0.0090 (17)	0.0011 (15)
C23	0.066 (2)	0.0791 (19)	0.078 (2)	−0.0034 (19)	0.0058 (19)	0.0283 (18)
C24	0.070 (2)	0.0465 (15)	0.117 (3)	−0.0008 (18)	−0.015 (2)	−0.0007 (19)
C25	0.0627 (18)	0.0582 (16)	0.0722 (19)	0.0106 (17)	0.0095 (18)	−0.0040 (16)
C26	0.0556 (17)	0.0552 (15)	0.0739 (19)	−0.0023 (17)	−0.0038 (18)	−0.0032 (16)
C27	0.0718 (19)	0.0519 (15)	0.0568 (16)	0.0080 (16)	0.0083 (18)	0.0032 (14)
C28	0.102 (3)	0.0570 (17)	0.085 (2)	0.018 (2)	−0.016 (2)	−0.0103 (18)
C29	0.085 (2)	0.0624 (17)	0.083 (2)	0.000 (2)	0.006 (2)	0.0221 (17)
C30	0.0629 (18)	0.0577 (16)	0.0572 (17)	0.0024 (17)	0.0026 (17)	0.0015 (15)
C31	0.080 (2)	0.0779 (18)	0.0681 (18)	−0.017 (2)	0.005 (2)	−0.0234 (17)
C32	0.0621 (18)	0.0638 (17)	0.0584 (17)	0.0028 (17)	0.0008 (17)	−0.0110 (15)
C33	0.085 (2)	0.0689 (19)	0.091 (2)	0.012 (2)	−0.012 (2)	−0.0114 (19)
C34	0.109 (3)	0.0729 (19)	0.095 (2)	−0.023 (2)	0.015 (3)	−0.013 (2)
C35	0.089 (2)	0.125 (3)	0.082 (2)	−0.034 (2)	0.013 (2)	−0.043 (2)
C36	0.073 (2)	0.146 (3)	0.071 (2)	0.032 (3)	−0.014 (2)	−0.023 (2)
C37	0.090 (2)	0.078 (2)	0.074 (2)	0.011 (2)	−0.005 (2)	−0.0125 (18)

### Geometric parameters (Å, °)

**Table:**

O1—N1	1.398 (3)	C17—C22	1.546 (4)
O1—N2	1.401 (3)	C18—C19	1.545 (3)
O2—C30	1.200 (3)	C18—H18A	0.9800
O3—C30	1.349 (3)	C19—C20	1.541 (3)
O3—C31	1.454 (3)	C19—H19A	0.9700
N1—C3	1.308 (3)	C19—H19B	0.9700
N2—C2	1.302 (4)	C20—C21	1.529 (4)
C1—C2	1.497 (4)	C20—C29	1.536 (4)
C1—C10	1.551 (4)	C20—C28	1.550 (4)
C1—H1A	0.9700	C21—C22	1.528 (4)
C1—H1B	0.9700	C21—H21A	0.9700
C2—C3	1.413 (4)	C21—H21B	0.9700
C3—C4	1.491 (4)	C22—H22A	0.9700
C4—C23	1.540 (4)	C22—H22B	0.9700
C4—C24	1.554 (4)	C23—H23A	0.9600
C4—C5	1.579 (3)	C23—H23B	0.9600
C5—C6	1.525 (4)	C23—H23C	0.9600
C5—C10	1.545 (4)	C24—H24A	0.9600
C5—H5A	0.9800	C24—H24B	0.9600
C6—C7	1.532 (3)	C24—H24C	0.9600
C6—H6A	0.9700	C25—H25A	0.9600
C6—H6B	0.9700	C25—H25B	0.9600
C7—C8	1.533 (3)	C25—H25C	0.9600
C7—H7A	0.9700	C26—H26A	0.9600
C7—H7B	0.9700	C26—H26B	0.9600
C8—C9	1.555 (4)	C26—H26C	0.9600
C8—C26	1.567 (3)	C27—H27A	0.9600
C8—C14	1.593 (3)	C27—H27B	0.9600
C9—C11	1.524 (3)	C27—H27C	0.9600
C9—C10	1.567 (3)	C28—H28A	0.9600
C9—H9A	0.9800	C28—H28B	0.9600
C10—C25	1.556 (4)	C28—H28C	0.9600
C11—C12	1.496 (3)	C29—H29A	0.9600
C11—H11A	0.9700	C29—H29B	0.9600
C11—H11B	0.9700	C29—H29C	0.9600
C12—C13	1.332 (4)	C31—C32	1.490 (4)
C12—H12A	0.9300	C31—H31A	0.9700
C13—C18	1.533 (3)	C31—H31B	0.9700
C13—C14	1.537 (3)	C32—C33	1.358 (4)
C14—C15	1.545 (4)	C32—C37	1.376 (4)
C14—C27	1.582 (3)	C33—C34	1.372 (5)
C15—C16	1.531 (3)	C33—H33A	0.9300
C15—H15A	0.9700	C34—C35	1.340 (5)
C15—H15B	0.9700	C34—H34A	0.9300
C16—C17	1.526 (4)	C35—C36	1.377 (5)
C16—H16A	0.9700	C35—H35A	0.9300
C16—H16B	0.9700	C36—C37	1.402 (5)
C17—C30	1.525 (4)	C36—H36A	0.9300
C17—C18	1.544 (4)	C37—H37A	0.9300
			
N1—O1—N2	110.42 (19)	C13—C18—H18A	107.0
C30—O3—C31	116.2 (2)	C17—C18—H18A	107.0
C3—N1—O1	105.3 (2)	C19—C18—H18A	107.0
C2—N2—O1	104.6 (2)	C20—C19—C18	114.0 (2)
C2—C1—C10	109.5 (2)	C20—C19—H19A	108.8
C2—C1—H1A	109.8	C18—C19—H19A	108.8
C10—C1—H1A	109.8	C20—C19—H19B	108.8
C2—C1—H1B	109.8	C18—C19—H19B	108.8
C10—C1—H1B	109.8	H19A—C19—H19B	107.6
H1A—C1—H1B	108.2	C21—C20—C29	111.9 (2)
N2—C2—C3	110.6 (2)	C21—C20—C19	108.4 (2)
N2—C2—C1	126.4 (2)	C29—C20—C19	111.4 (2)
C3—C2—C1	123.0 (2)	C21—C20—C28	108.6 (2)
N1—C3—C2	109.1 (2)	C29—C20—C28	107.7 (2)
N1—C3—C4	125.1 (2)	C19—C20—C28	108.9 (2)
C2—C3—C4	125.8 (2)	C22—C21—C20	112.2 (2)
C3—C4—C23	108.3 (2)	C22—C21—H21A	109.2
C3—C4—C24	109.2 (2)	C20—C21—H21A	109.2
C23—C4—C24	108.6 (2)	C22—C21—H21B	109.2
C3—C4—C5	108.5 (2)	C20—C21—H21B	109.2
C23—C4—C5	108.5 (2)	H21A—C21—H21B	107.9
C24—C4—C5	113.7 (2)	C21—C22—C17	112.5 (2)
C6—C5—C10	111.5 (2)	C21—C22—H22A	109.1
C6—C5—C4	111.9 (2)	C17—C22—H22A	109.1
C10—C5—C4	117.8 (2)	C21—C22—H22B	109.1
C6—C5—H5A	104.8	C17—C22—H22B	109.1
C10—C5—H5A	104.8	H22A—C22—H22B	107.8
C4—C5—H5A	104.8	C4—C23—H23A	109.5
C5—C6—C7	110.2 (2)	C4—C23—H23B	109.5
C5—C6—H6A	109.6	H23A—C23—H23B	109.5
C7—C6—H6A	109.6	C4—C23—H23C	109.5
C5—C6—H6B	109.6	H23A—C23—H23C	109.5
C7—C6—H6B	109.6	H23B—C23—H23C	109.5
H6A—C6—H6B	108.1	C4—C24—H24A	109.5
C6—C7—C8	113.8 (2)	C4—C24—H24B	109.5
C6—C7—H7A	108.8	H24A—C24—H24B	109.5
C8—C7—H7A	108.8	C4—C24—H24C	109.5
C6—C7—H7B	108.8	H24A—C24—H24C	109.5
C8—C7—H7B	108.8	H24B—C24—H24C	109.5
H7A—C7—H7B	107.7	C10—C25—H25A	109.5
C7—C8—C9	110.5 (2)	C10—C25—H25B	109.5
C7—C8—C26	107.9 (2)	H25A—C25—H25B	109.5
C9—C8—C26	111.2 (2)	C10—C25—H25C	109.5
C7—C8—C14	110.2 (2)	H25A—C25—H25C	109.5
C9—C8—C14	108.48 (19)	H25B—C25—H25C	109.5
C26—C8—C14	108.5 (2)	C8—C26—H26A	109.5
C11—C9—C8	109.3 (2)	C8—C26—H26B	109.5
C11—C9—C10	114.1 (2)	H26A—C26—H26B	109.5
C8—C9—C10	117.6 (2)	C8—C26—H26C	109.5
C11—C9—H9A	104.8	H26A—C26—H26C	109.5
C8—C9—H9A	104.8	H26B—C26—H26C	109.5
C10—C9—H9A	104.8	C14—C27—H27A	109.5
C5—C10—C1	108.1 (2)	C14—C27—H27B	109.5
C5—C10—C25	111.6 (2)	H27A—C27—H27B	109.5
C1—C10—C25	107.5 (2)	C14—C27—H27C	109.5
C5—C10—C9	109.2 (2)	H27A—C27—H27C	109.5
C1—C10—C9	106.51 (19)	H27B—C27—H27C	109.5
C25—C10—C9	113.7 (2)	C20—C28—H28A	109.5
C12—C11—C9	113.9 (2)	C20—C28—H28B	109.5
C12—C11—H11A	108.8	H28A—C28—H28B	109.5
C9—C11—H11A	108.8	C20—C28—H28C	109.5
C12—C11—H11B	108.8	H28A—C28—H28C	109.5
C9—C11—H11B	108.8	H28B—C28—H28C	109.5
H11A—C11—H11B	107.7	C20—C29—H29A	109.5
C13—C12—C11	126.4 (2)	C20—C29—H29B	109.5
C13—C12—H12A	116.8	H29A—C29—H29B	109.5
C11—C12—H12A	116.8	C20—C29—H29C	109.5
C12—C13—C18	119.0 (2)	H29A—C29—H29C	109.5
C12—C13—C14	120.4 (2)	H29B—C29—H29C	109.5
C18—C13—C14	120.6 (2)	O2—C30—O3	121.8 (3)
C13—C14—C15	112.1 (2)	O2—C30—C17	126.4 (3)
C13—C14—C27	107.1 (2)	O3—C30—C17	111.8 (2)
C15—C14—C27	107.3 (2)	O3—C31—C32	106.9 (2)
C13—C14—C8	108.9 (2)	O3—C31—H31A	110.3
C15—C14—C8	109.7 (2)	C32—C31—H31A	110.3
C27—C14—C8	111.70 (19)	O3—C31—H31B	110.3
C16—C15—C14	114.4 (2)	C32—C31—H31B	110.3
C16—C15—H15A	108.7	H31A—C31—H31B	108.6
C14—C15—H15A	108.7	C33—C32—C37	118.2 (3)
C16—C15—H15B	108.7	C33—C32—C31	121.2 (3)
C14—C15—H15B	108.7	C37—C32—C31	120.6 (3)
H15A—C15—H15B	107.6	C32—C33—C34	122.1 (3)
C17—C16—C15	112.9 (2)	C32—C33—H33A	118.9
C17—C16—H16A	109.0	C34—C33—H33A	118.9
C15—C16—H16A	109.0	C35—C34—C33	118.9 (3)
C17—C16—H16B	109.0	C35—C34—H34A	120.6
C15—C16—H16B	109.0	C33—C34—H34A	120.6
H16A—C16—H16B	107.8	C34—C35—C36	122.5 (4)
C30—C17—C16	111.6 (2)	C34—C35—H35A	118.7
C30—C17—C18	108.4 (2)	C36—C35—H35A	118.7
C16—C17—C18	109.5 (2)	C35—C36—C37	117.1 (3)
C30—C17—C22	104.7 (2)	C35—C36—H36A	121.5
C16—C17—C22	111.2 (2)	C37—C36—H36A	121.5
C18—C17—C22	111.3 (2)	C32—C37—C36	121.2 (3)
C13—C18—C17	112.1 (2)	C32—C37—H37A	119.4
C13—C18—C19	111.2 (2)	C36—C37—H37A	119.4
C17—C18—C19	112.1 (2)		
			
N2—O1—N1—C3	−0.9 (3)	C12—C13—C14—C8	−25.8 (3)
N1—O1—N2—C2	1.1 (3)	C18—C13—C14—C8	154.5 (2)
O1—N2—C2—C3	−0.9 (3)	C7—C8—C14—C13	176.9 (2)
O1—N2—C2—C1	−178.5 (2)	C9—C8—C14—C13	55.8 (3)
C10—C1—C2—N2	150.4 (3)	C26—C8—C14—C13	−65.1 (3)
C10—C1—C2—C3	−26.9 (4)	C7—C8—C14—C15	−60.0 (3)
O1—N1—C3—C2	0.3 (3)	C9—C8—C14—C15	178.9 (2)
O1—N1—C3—C4	−179.5 (2)	C26—C8—C14—C15	58.0 (3)
N2—C2—C3—N1	0.4 (3)	C7—C8—C14—C27	58.9 (3)
C1—C2—C3—N1	178.1 (2)	C9—C8—C14—C27	−62.3 (3)
N2—C2—C3—C4	−179.8 (3)	C26—C8—C14—C27	176.8 (2)
C1—C2—C3—C4	−2.1 (4)	C13—C14—C15—C16	−38.5 (3)
N1—C3—C4—C23	63.8 (3)	C27—C14—C15—C16	78.8 (3)
C2—C3—C4—C23	−116.0 (3)	C8—C14—C15—C16	−159.6 (2)
N1—C3—C4—C24	−54.2 (4)	C14—C15—C16—C17	54.7 (3)
C2—C3—C4—C24	125.9 (3)	C15—C16—C17—C30	59.8 (3)
N1—C3—C4—C5	−178.6 (2)	C15—C16—C17—C18	−60.3 (3)
C2—C3—C4—C5	1.6 (4)	C15—C16—C17—C22	176.3 (2)
C3—C4—C5—C6	160.9 (2)	C12—C13—C18—C17	140.0 (2)
C23—C4—C5—C6	−81.6 (3)	C14—C13—C18—C17	−40.2 (3)
C24—C4—C5—C6	39.2 (3)	C12—C13—C18—C19	−93.6 (3)
C3—C4—C5—C10	29.8 (3)	C14—C13—C18—C19	86.1 (3)
C23—C4—C5—C10	147.2 (2)	C30—C17—C18—C13	−70.7 (3)
C24—C4—C5—C10	−91.9 (3)	C16—C17—C18—C13	51.4 (3)
C10—C5—C6—C7	−61.6 (3)	C22—C17—C18—C13	174.7 (2)
C4—C5—C6—C7	164.1 (2)	C30—C17—C18—C19	163.4 (2)
C5—C6—C7—C8	58.4 (3)	C16—C17—C18—C19	−74.6 (3)
C6—C7—C8—C9	−47.9 (3)	C22—C17—C18—C19	48.8 (3)
C6—C7—C8—C26	73.9 (3)	C13—C18—C19—C20	−178.7 (2)
C6—C7—C8—C14	−167.8 (2)	C17—C18—C19—C20	−52.3 (3)
C7—C8—C9—C11	175.45 (19)	C18—C19—C20—C21	55.3 (3)
C26—C8—C9—C11	55.6 (3)	C18—C19—C20—C29	−68.2 (3)
C14—C8—C9—C11	−63.7 (2)	C18—C19—C20—C28	173.2 (2)
C7—C8—C9—C10	43.3 (3)	C29—C20—C21—C22	66.0 (3)
C26—C8—C9—C10	−76.5 (3)	C19—C20—C21—C22	−57.2 (3)
C14—C8—C9—C10	164.2 (2)	C28—C20—C21—C22	−175.3 (2)
C6—C5—C10—C1	170.1 (2)	C20—C21—C22—C17	57.3 (3)
C4—C5—C10—C1	−58.6 (3)	C30—C17—C22—C21	−168.8 (2)
C6—C5—C10—C25	−72.0 (3)	C16—C17—C22—C21	70.6 (3)
C4—C5—C10—C25	59.3 (3)	C18—C17—C22—C21	−51.8 (3)
C6—C5—C10—C9	54.6 (3)	C31—O3—C30—O2	0.1 (4)
C4—C5—C10—C9	−174.1 (2)	C31—O3—C30—C17	178.8 (2)
C2—C1—C10—C5	53.2 (3)	C16—C17—C30—O2	−145.9 (3)
C2—C1—C10—C25	−67.3 (3)	C18—C17—C30—O2	−25.2 (4)
C2—C1—C10—C9	170.4 (2)	C22—C17—C30—O2	93.7 (3)
C11—C9—C10—C5	−176.7 (2)	C16—C17—C30—O3	35.4 (3)
C8—C9—C10—C5	−46.8 (3)	C18—C17—C30—O3	156.1 (2)
C11—C9—C10—C1	66.8 (3)	C22—C17—C30—O3	−85.0 (3)
C8—C9—C10—C1	−163.3 (2)	C30—O3—C31—C32	174.1 (2)
C11—C9—C10—C25	−51.4 (3)	O3—C31—C32—C33	−111.0 (3)
C8—C9—C10—C25	78.5 (3)	O3—C31—C32—C37	67.0 (3)
C8—C9—C11—C12	39.5 (3)	C37—C32—C33—C34	0.5 (5)
C10—C9—C11—C12	173.5 (2)	C31—C32—C33—C34	178.6 (3)
C9—C11—C12—C13	−8.8 (4)	C32—C33—C34—C35	−0.7 (5)
C11—C12—C13—C18	−178.2 (2)	C33—C34—C35—C36	1.7 (5)
C11—C12—C13—C14	2.1 (4)	C34—C35—C36—C37	−2.3 (5)
C12—C13—C14—C15	−147.4 (2)	C33—C32—C37—C36	−1.1 (4)
C18—C13—C14—C15	32.9 (3)	C31—C32—C37—C36	−179.3 (3)
C12—C13—C14—C27	95.2 (3)	C35—C36—C37—C32	2.0 (5)
C18—C13—C14—C27	−84.6 (3)		
